# Military Injuries—Understanding Posttraumatic Epilepsy, Health, and Quality-of-Life Effects of Caregiving: Protocol for a Longitudinal Mixed Methods Observational Study

**DOI:** 10.2196/30975

**Published:** 2022-01-05

**Authors:** Erin D Bouldin, Roxana Delgado, Kimberly Peacock, Willie Hale, Ali Roghani, Amira Y Trevino, Mikayla Viny, David W Wetter, Mary Jo Pugh

**Affiliations:** 1 Department of Health and Exercise Science Appalchian State University Boone, NC United States; 2 Department of Medicine University of Texas Health Science Center at San Antonio San Antonio, TX United States; 3 Department of Psychology University of Texas at San Antonio San Antonio, TX United States; 4 Department of Internal Medicine University of Utah School of Medicine Salt Lake City, UT United States; 5 Department of Population Health Sciences University of Utah School of Medicine Salt Lake City, UT United States; 6 Informatics, Decision-Enhancement, and Analytic Sciences Center Veterans Affairs Salt Lake City Health Care System Salt Lake City, UT United States

**Keywords:** epilepsy, military personnel, veterans, caregiver, traumatic brain injury, quality of life, health status, longitudinal studies, ecologic momentary assessment, qualitative research

## Abstract

**Background:**

Veterans with posttraumatic epilepsy (PTE), particularly those with comorbidities associated with epilepsy or traumatic brain injury (TBI), have poorer health status and higher symptom burden than their peers without PTE. One area that has been particularly poorly studied is that of the role of caregivers in the health of veterans with PTE and the impact caring for someone with PTE has on the caregivers themselves.

**Objective:**

In this study, we aim to address the following: describe and compare the health and quality of life of veterans and caregivers of veterans with and without PTE; evaluate the change in available supports and unmet needs for services among caregivers of post-9/11 veterans with PTE over a 2-year period and to compare support and unmet needs with those without PTE; and identify veteran and caregiver characteristics associated with the 2-year health trajectories of caregivers and veterans with PTE compared with veterans without PTE.

**Methods:**

We conducted a prospective cohort study of the health and quality of life among 4 groups of veterans and their caregivers: veterans with PTE, nontraumatic epilepsy, TBI only, and neither epilepsy nor TBI. We will recruit participants from previous related studies and collect information about both the veterans and their primary informal caregivers on health, quality of life, unmet needs for care, PTE and TBI symptoms and treatment, relationship, and caregiver experience. Data sources will include existing data supplemented with primary data, such as survey data collected at baseline, intermittent brief reporting using ecological momentary assessment, and qualitative interviews. We will make both cross-sectional and longitudinal comparisons, using veteran-caregiver dyads, along with qualitative findings to better understand risk and promotive factors for quality of life and health among veterans and caregivers, as well as the bidirectional impact of caregivers and care recipients on one another.

**Results:**

This study was approved by the institutional review boards of the University of Utah and Salt Lake City Veterans Affairs and is under review by the Human Research Protection Office of the United States Army Medical Research and Development Command. The Service Member, Veteran, and Caregiver Community Stakeholders Group has been formed and the study questionnaire will be finalized once the panel reviews it. We anticipate the start of recruitment and primary data collection by January 2022.

**Conclusions:**

New national initiatives aim to incorporate the caregiver into the veteran’s treatment plan; however, we know little about the impact of caregiving—both positive and negative—on the caregivers themselves and on the veterans for whom they provide care. We will identify specific needs in this understudied population, which will inform clinicians, patients, families, and policy makers about the specific impact and needs to equip caregivers in caring for veterans at home.

**International Registered Report Identifier (IRRID):**

PRR1-10.2196/30975

## Introduction

### Background

Epilepsy is a substantial concern among United States service members and veterans (hereafter referred to as *veterans* as service members ultimately become veterans). Among post-9/11 veterans, prior studies have found a prevalence of epilepsy of approximating 10.6/1000 [1]. In particular, posttraumatic epilepsy (PTE), which occurs following a traumatic brain injury (TBI), is more common among veterans who served during Operation Enduring Freedom or Operation Iraqi Freedom (post-9/11) because of the higher incidence of TBI and blast injuries compared with earlier conflicts [1-3]. The annual cost of epilepsy care in the United States has been estimated at US $12.5 billion [4], with higher costs for those newly diagnosed, those with seizures refractory to anticonvulsant medication treatment, and those with comorbid health conditions [5,6]. Indirect costs (eg, caretaker’s time away from work) comprise most of the total cost of epilepsy care [7,8]. Estimates of caregiver-related costs based on self-report are high [9].

Poorly controlled epilepsy is associated with injury, disability, mortality, and poor quality of life [10-15]. Veterans with epilepsy have also reported role limitations caused by impairments related to physical, mental, emotional, and social functions [16,17]. Of the post-9/11 deployed veterans, 11% to 23% experienced TBI, most of which were mild TBIs (mTBIs) [2,18-21]. Even among those with mTBI, the risk of developing PTE is elevated (adjusted odds ratio 1.28, 95% CI 1.07-1.53) [1]. With hundreds of thousands of veterans exposed to mTBI, even a small elevated risk has the potential to impact thousands of individuals, caregivers, and families.

Our understanding of epilepsy and the impact of PTE in post-9/11 combat veterans is limited. Data from the Department of Veterans Affairs’ (VA) comprehensive TBI evaluation have shown that veterans with PTE have significantly higher cognitive, affective, somatosensory, and vestibular symptom burdens than those with mTBI only. A survey-based study found that veterans with PTE had significantly lower quality of life, social support, and family resilience scores compared with those with epilepsy, mTBI, and control participants with neither TBI nor epilepsy [22]. Given the relatively young age of post-9/11 veterans, the costs of care (both personal and financial) may profoundly affect the lives of these veterans, their caregivers or families, and the health care systems on which they depend for care.

Informal caregivers—family and friends who provide assistance for people living with chronic health conditions or disabilities living in the community—in general, have significantly higher levels of stress, depression, and lower levels of physical health than noncaregivers [23-25]. Although positive effects of caregiving have been reported [26], caregiving can negatively influence caregiver health, and these effects are likely unique to specific caregiver populations. The effects of caregiving for veterans with PTE are understudied. Qualitative interviews indicated that caregivers experience stress, anxiety, and secondary posttraumatic stress disorder because of frequent *seizure or suicide watch* when caring for a veteran with PTE. These data suggest unmet needs for support and services owing to a lack of tailored programs and support for veterans with PTE and their caregivers. Most prior studies that have assessed the dyadic relationship between caregivers and care recipients have focused on children with epilepsy; therefore, assessments of the quality of life for veterans with PTE and their caregivers are needed.

The available evidence regarding military caregiving suggests that these caregivers may experience a health decline for a longer period than any other caregiver population [27-29]. The young age and long life span of veterans with TBI, PTE, and other catastrophic injuries make this population unique in light of the existing literature, which has focused on family caregivers of patients in hospice or patients with a diagnosis of cancer, dementia, Parkinson disease, Alzheimer disease, and other diseases or disabilities [30-32]. The full range of experiences and needs of caregivers of veterans with TBI in general and of those with PTE in particular has not been fully explored. For example, other researchers have highlighted the need to understand family and relationship quality [33], caregiver resilience [34], and positive aspects of the caregiver experience [35] to support veterans with TBI and their caregivers.

Given these gaps in knowledge, the purpose of this study is to longitudinally examine factors associated with quality of life and health outcomes for veterans with epilepsy or PTE and their caregivers. To develop appropriate interventions to improve health outcomes for veterans and their families, we must understand the veteran-caregiver dynamics.

### Hypotheses and Objectives

On the basis of the existing literature and our preliminary studies, we hypothesize that caregivers of veterans with PTE encounter unique challenges in their caregiving role, resulting in more intense caregiving and a higher burden than caregivers of other post-9/11 US veterans, including those with TBI only or nontraumatic epilepsy (NTE). Furthermore, we hypothesize that caregivers of veterans with PTE and associated epilepsy comorbidities (eg, depression and headache) have a higher burden and poorer health than caregivers of veterans with PTE but without associated comorbidities. Therefore, we expect that the health and burden trajectories of caregivers of veterans with PTE will differ from caregivers of veterans without PTE and that there will be distinctions between these groups based on whether the veteran has associated comorbidities. Finally, we hypothesize that the caregiver’s burden and own health trajectory will influence the health status of the veteran at baseline and their health trajectories during follow-up.

This study will evaluate the health, well-being, and quality of life of veterans and their caregivers with the following aims: (1) describe and compare the health and quality of life of veterans and caregivers of veterans with and without PTE; (2) evaluate the change in available support and unmet needs for services among caregivers of post-9/11 veterans with PTE over a 2-year period and compare supports and unmet needs with those without PTE; and (3) identify veteran and caregiver characteristics associated with the 2-year health trajectories of caregivers and veterans with PTE compared with veterans without PTE.

## Methods

### Conceptual Framework

**Figure 1:**
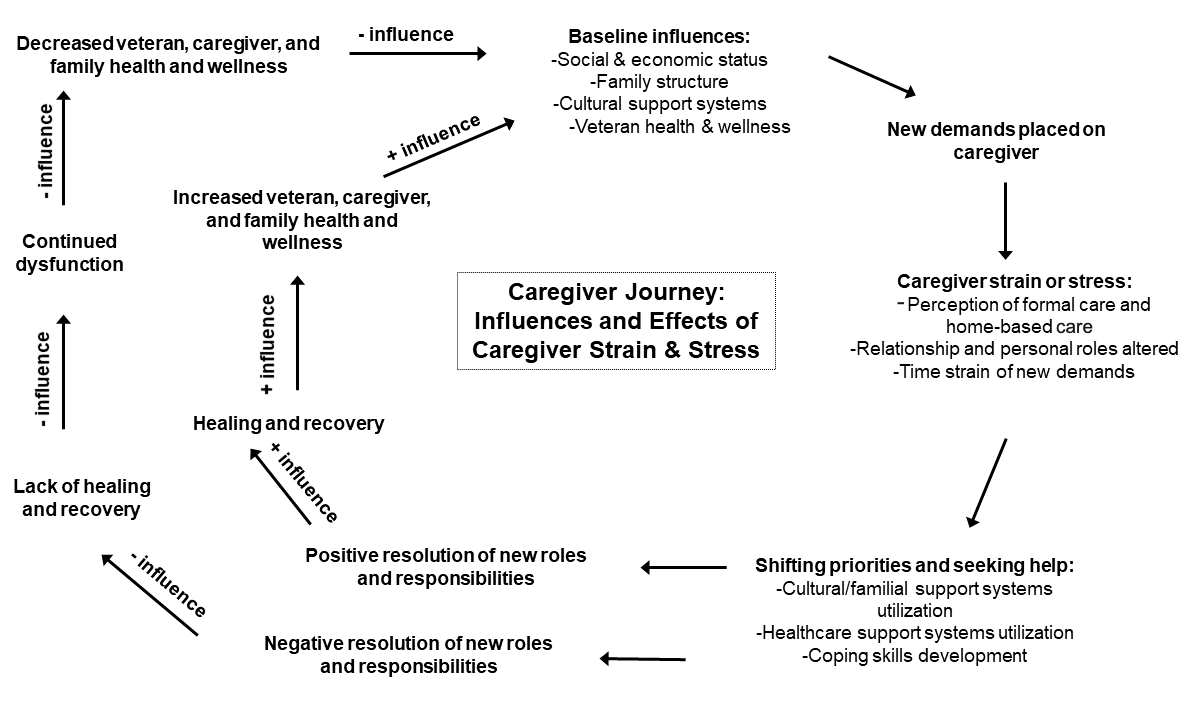
Study conceptual framework modified and adapted from Raina et al [36] and the Military and Veteran Caregiver Map [37].

This study is guided by a biopsychosocial model of caregiving [36] and the Military and Veteran Caregiver Experience Map [37]. Figure 1 [36,37] is an adaptation of the Military and Veteran Caregiver Experience Map developed over the course of several years by the Elizabeth Dole Foundation and military and veteran caregiver (MVC) stakeholders [37] and Raina’s synthesis of theoretical frameworks for the impact of caregiving [36]. The model posits that baseline characteristics (ie, social and economic status, family structure, cultural support systems, and veteran health and wellness) influence the caregiver’s ability to meet the new demands of caregiving, which may lead to caregiver stress or strain depending on the extent to which their current identity and roles are altered by caregiving requirements. Over time, the caregiver may shift priorities and seek help within current social or family circles or the health care system and may develop new coping skills. When the caregiver is unable to shift priorities or obtain the needed support within social relationships or the health care system, there is a negative impact on caregiver well-being, continued dysfunction, and diminished veteran, caregiver, and family function, which can then lead to a negative impact on baseline influences and a negative spiral of health and well-being. When the caregiver is able to adapt or cope with new roles and responsibilities, there is a positive impact on veterans, caregivers, and family well-being, as well as a positive impact on baseline characteristics with a positive spiral for health and well-being for veterans, caregivers, and families. Survey and ecological momentary assessment (EMA) data collection will obtain data that address each component of this model and use modeling techniques to explicate the trajectories of veteran and caregiver health or well-being and quality of life.

### Study Design

We will conduct a prospective cohort study of the health and quality of life among 4 groups of veterans and their caregivers: (1) PTE, (2) NTE, (3) TBI only, and (4) neither epilepsy nor TBI. We will recruit participants from previous studies of PTE or TBI and MVC outcomes conducted by Dr Pugh and Dr Delgado; specifically, we will sample from among respondents who agreed to be contacted for future research studies. Data sources will include existing study data from these prior projects supplemented with primary data collected for this study in 2 forms: (1) survey data collected at baseline; (2) daily brief reporting using EMA [38] over the course of 30 days, with the option to continue this data collection for up to 2 years; and (3) qualitative data collected via interviews.

### Service Member, Veteran, and Caregiver Community Stakeholders Group

We established a Service Member, Veteran, and Caregiver Community Stakeholders Group for this study, which consists of 3 veterans with the goal of having 1 with TBI, 1 with epilepsy, and 1 without TBI or epilepsy, and 3 caregivers of veterans with TBI or epilepsy. We recruited locally using existing relationships and used state and national service members, veterans, and caregiver organizations as recruitment sources (eg, Wounded Warrior Programs, American Red Cross’ Military and Veteran Caregiver Network, and Iraq and Afghanistan Veterans of America). This group will help identify special issues in addressing veteran and caregiver needs and ensure that our approach is consistent with community needs and values. Members will receive stipends for their participation. We will consult with the community stakeholders to finalize the veteran and caregiver surveys and EMA items, and we will discuss findings with them to assure our interpretation of the results resonate with their experiences and understanding of living with and providing care to veterans with TBI and PTE. Full community stakeholders group meetings are planned quarterly throughout the study period.

### Study Population

We will recruit veteran and caregivers for this study from among a group of participants who agreed to be recontacted from the Veterans Posttraumatic Epilepsy Study (VPES; 189/210, 90% response rate in follow-up interviews), the pilot TBI Caregiver Study, and the Military and Veteran Caregiver Health and Well-being (MVCHW) study. We will request participation from the primary caregiver for VPES participants, veterans for the MVCHW, and recontact both veterans and the primary caregiver from the pilot TBI Caregiver Study. The group of veterans with neither TBI nor epilepsy includes people with primary mental health comorbidities or devastating diseases such as amyotrophic lateral sclerosis. These groups will allow us to examine variations in caregiver burden, stressors, resources used, and unmet needs based on epilepsy and comorbidity patterns.

### Inclusion and Exclusion Criteria

As we are recruiting from 3 prior studies, the inclusion criteria and sampling frame for each of these studies vary slightly and are summarized below.

The VPES included veterans who were deployed in support of post-9/11 conflicts and received ≥2 years of VA care between fiscal year 2002 and fiscal year 2014 with at least one of those years after 2007, when VA began mandatory TBI screening*. Epilepsy* was identified using our validated algorithm (International Classification of Disease-9 or -10 diagnosis of epilepsy or convulsion and subsequent use of antiseizure medication) [1], with subsequent validation by medical chart abstraction. TBI was identified using the International Classification of Disease-9 or -10 algorithm developed by the Armed Forces Health Surveillance System [39], and self-reported lifetime TBI history based on the Ohio State TBI Identification measure [40]. Veterans with PTE, NTE, TBI, and controls (veterans with neither epilepsy nor TBI) were randomly selected for survey administration. Only those veterans who responded and agreed to be recontacted for future research and also reported having a caregiver were included in this study (269/326, 82.5% reported having a caregiver).

The MVCHW study recruited participants using email invitations sent out by MVC support organizations such as the Elizabeth Dole Foundation and Hearts of Valor in April 2017. Although no denominator was provided to the study team, 476 individuals responded and completed the web-based survey within a 3-month period without any incentive. Caregivers self-reported the types of conditions for which they provided care.

The pilot TBI Caregiver Study invited 186 veterans who were diagnosed with penetrating TBI (per the Armed Forces Health Surveillance System algorithm) and their caregivers via email. Upon validation of TBI severity using the Ohio State TBI Identification measure [40], it was found that all had moderate or severe TBI. Of the 186 invites, 66 (35.4%) veterans and caregivers responded, and 65 (34.9%) dyads agreed to be recontacted for future research.

For this study, we will use data on TBI and epilepsy history to classify all veteran-caregiver dyads into 1 of the 4 study groups. Only caregivers aged >18 years will be included. Although we will inquire about caregiving provided by children, we will not engage minors in the study.

### Sample Size

We expect to achieve a response rate of 75% given that we will sample from among veterans who have already participated in a study and expressed a willingness to be recontacted. We also expect a high participation rate among caregivers, as the veteran has already participated and presumably will have told the caregiver about the study. We plan to enroll 97 veterans with PTE, 45 with NTE, 323 with TBI only, and 145 with no PTE and no TBI and their caregivers, totaling 610 veterans and 610 caregivers. Table 1 shows the number of participants in each of the 4 study groups.

**Table 1:** Anticipated sample size from each recruitment source study, assuming a 75% response rate (N=610).

Recruitment source	Number of veterans, n (%)				
	With posttraumatic epilepsy (n=97)	With nontraumatic epilepsy (n=45)	With TBI^a^ only (n=323)	With no epilepsy and no TBI (n=145)	Total (N=610)
Veterans Posttraumatic Epilepsy Study	42 (43.3)	43 (95.6)	88 (27.2)	37 (25.5)	210 (34.4)
Military and Veteran Caregiver Health and Well-being Study	36 (37.1)	2 (4.4)	204 (63.1)	108 (74.4)	350 (57.3)
Pilot TBI Caregiver Study	19 (19.6)	—^b^	31 (9.5)	—	50 (8.2)

^a^TBI: traumatic brain injury.

^b^Respondents in this category were not included in the study.

We plan to compare veterans or caregivers of veterans with PTE to those with (1) NTE, (2) TBI only, and (3) no epilepsy and no TBI. We anticipate adequate statistical power for planned analyses using the survey data. For example, we have >80% power to detect a 2-point difference between veterans with PTE and those with NTE, the minimum size considered clinically meaningful for the Veterans RAND 12-Item Health Survey, with an SD as high as 4.25, even if we account for the correlation between veterans and caregivers (assumptions: cluster size of 2, intraclass correlation coefficient of 0.8 between veterans and caregivers, and α=.05) [41]. We assume similar variability among caregivers and therefore also anticipate adequate power when comparing the experiences of caregivers of veterans with PTE and NTE. We may not have adequate power to detect differences between groups when making comparisons based on health trajectory groups, given that we anticipate 3 groups, 2 of which may be moderate to small. However, our qualitative analysis will allow us an additional perspective in comparing PTE and NTE.

### Sampling

**Figure 2:**
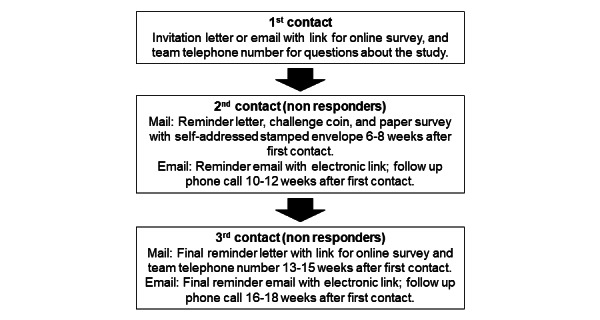
Process for contacting potential study participants based on Dillman Tailored Design Method for mail and web-based surveys.

We will collect survey data from both veterans and caregivers following the Dillman Tailored Design Method for mail and web-based surveys to minimize nonresponse errors [42]. This approach involves a combination of data collection strategies to achieve a higher response rate. Our specific strategy will involve either mail or email, depending on what contact information we have for the veteran and caregiver, and is outlined in Figure 2.

To encourage participation, we will provide an incentive of US $25 for the baseline survey and US $2 per response for the first 30 days of EMA data collection. In addition, we will offer incentives for the remainder of the 2-year EMA period by entering participants in a drawing for 5 US $500 gift cards and 10 US $100 gift cards per quarter for veterans and caregivers. This approach was used in a previous study and improved response rates by over 60% (from 30% to 48%).

### Data Collection

We will collect survey data from both veterans and their primary caregivers at baseline. Our caregiver survey was modeled based on the RAND Military Caregivers Study [23], the Behavioral Risk Factor Surveillance System (BRFSS) Caregiver Module [43], and the National Alliance for Caregiving survey [44]. The goal is to develop a comprehensive survey that assesses various aspects of the respondent’s health, quality of life, and service needs in a reasonable amount of time (≤30 minutes). The Service Member, Veteran, and Caregiver Community Stakeholders Group will review the baseline survey and recommend changes before it is fielded. Veterans and caregivers who complete the baseline survey will be invited to participate in EMA data collection for up to 2 years.

We will mail or email information on how to use the LifeData EMA platform to those who agreed to participate in the EMA. Participants will also be given contact information of study personnel who can assist the participant with the platform setup. Once set up, each participant will receive approximately 2 prompts daily to respond to for 30 days. The EMA will capture different measures daily. Participants can also add information to the EMA platform on concerns or unmet needs at any time using a *pull* mechanism where they initiate the data collection at the time of their choosing. Participants will receive US $2 per assessment completed for 30 days and also be eligible for drawings of larger incentives for continued EMA participation. The EMA platform (LifeData) is Health Insurance Portability and Accountability Act compliant and includes iOS, Android, and web-based platforms that can be used depending on the preference of the participant [45].

We will conduct qualitative interviews based on extreme variation sampling of results from early EMA data. Our descriptive phenomenological approach will be guided by the following research questions: (1) (Veteran) What are the epilepsy and TBI characteristics (ie, exposure, mechanism, and history) of veterans in a subsample of the cohort? (2) (Caregiver) How do veteran caregivers experience and perceive health, and what are the characteristics and unique factors that affect their overall health?

The goal of the qualitative component of this mixed methods study is to describe the insights and experiences of participants and to identify important elements of caregiving in this particular group that will help develop strategies for preventive care.

### Measures

#### Overview

We will classify veterans as having epilepsy or TBI using procedures consistent with the International League Against Epilepsy [46]. Two of the source cohorts were identified using VA health system data (VPES, TBI caregiver), where epilepsy was identified using a reliable epilepsy algorithm [1] and confirmed using medical chart abstraction; lifetime TBI was identified using the Ohio State University TBI Identification measure [40]. TBI severity and frequency is defined using the Ohio State University TBI measure, and PTE is defined as probable and possible PTE based on the US National Institute of Neurological Disorders and Stroke PTE screening measure. The third cohort includes the MVCHW cohort. Veterans from this study will complete PTE and TBI screening measures to have the same reliable classifications.

The primary method of assessing comorbidity, medical, and psychiatric conditions over time will be self-reported by veterans and their caregivers using survey and EMA methods. Although self-reported conditions have some limitations, studies including the BRFSS, the Millennium Cohort Study, and the Large Health Survey of Veterans routinely use these measures to gauge population health [47-49]. Whereas sensitivity varies by condition, the specificity was >80% for all conditions included in the BRFSS [47]. Sensitivity and specificity are higher when longer periods of observation in medical records are used [48]. Thus, this method is appropriate for this prospective observational study. We will also validate PTE, TBI, and baseline health status using measures in our baseline survey. This will enable us to classify respondents as having probable or suspected PTE (or TBI).

#### Baseline Survey

We will measure caregiver burden using the short version of the Zarit Burden Interview, a 12-item caregiver inventory [50]. Responses for each item will be measured using a 5-point Likert scale ranging from 0 (never) to 4 (nearly always), with scores summed to create an overall burden score. A score >16 on caregiver burden has been shown to reflect a clinically significant burden, which will be used as a dichotomous measure of high overall burden in this study. The National Alliance for Caregiving survey developed a level of care index that combines information about the number of activities of daily living and instrumental activities of daily living a caregiver performs with the amount of time a caregiver spends providing care [44]. We will use this measure to represent caregiving intensity in this study.

We will use the Veterans RAND version of the Medical Outcomes Survey Short-Form, a 12- item survey, to measure physical and mental health among both veterans and caregivers [51]. Each item will be rated on a Likert scale ranging from 0 (worst) to 4 (best). We will transform the scores using standard-based scoring [52]. A difference of 2 to 3 points is considered clinically important [53]. We will also use the presence of self-reported comorbid conditions (eg, chronic health conditions, pain, substance use disorder, and posttraumatic stress disorder) to evaluate veteran and caregiver health status and changes in health over time.

We will use the 4 items from the BRFSS Healthy Days Core Module to evaluate the quality of life [54]. These items assess overall health (from poor to excellent) and the number of days in the past month on which the respondent’s physical and mental health were not good, along with the number of days poor physical or mental health limited the respondent’s usual activities. We will create dichotomous variables for fair or poor health (vs excellent, very good, or good) and for frequent physical distress, mental distress, and activity limitation, defined as distress or limitations experienced for ≥14 days in the past 30 days [55].

We will adapt a caregiver needs assessment developed by O’Malley and Qualls [56] that evaluates both current service use, current needs, and reasons that services are not being used. The assessment includes 10 caregiver support items (eg, counseling, respite care, and support group), 12 care recipient support items (eg, help with housekeeping, home adaptation, and financial assistance), and 9 types of information needed (eg, list of local caregiving resources and information about long-term care planning). We may limit these items in consultation with the Service Member, Veteran, and Caregiver Community Stakeholders Group to keep the survey at a manageable length.

In addition to the main outcome measures described above, we will collect a wealth of additional information about veterans and caregivers, including validated and commonly used measures of disability status, social support, stress, thriving, caregiver-care recipient relationship, challenging behaviors, personal and family information, and socioeconomic status (Table 2).

**Table 2:** Domains and constructs covered in baseline veteran and caregiver surveys, and items used to assess each domain.

Domain and construct			Measure or item source
**Both veteran** **and caregiver survey**			
	Relationship between veteran and caregiver	Behavioral Risk Factor Surveillance System caregiver module [43] Dyadic Relationship Scale [57] Family Resilience Scale for Veterans [58]	
	Length of care	Modified Behavioral Risk Factor Surveillance System. caregiver module [43]	
	Caregiving tasks	Modified NAC^a^ Questionnaire [44]	
	Amount of care	NAC Questionnaire [44]	
	Primary caregiver	Modified NAC Questionnaire [44]	
	Social support	Perceived social support from the Veterans Health Study [59]	
	Health status	The Veterans RAND-12 item Health Survey [51]	
	Sleep	Insomnia Severity Index [60]	
	Traumatic brain injury screener	Ohio State University Traumatic Brain Injury Identification Method [40,61]	
	Service use	Modified O’Malley Measure of Caregiver Service Use [56]	
	Stress	Perceived Stress Scale-14 [62]	
	Thriving	Brief Inventory of Thriving [63]	
	Loneliness	Three-Item Loneliness Scale [64]	
	COVID-19	Lived Experience of Epilepsy: Patient and Caregiver Perspectives Study [65] Beach Questionnaire [66]	
	Anxiety	Generalized Anxiety Disorder 2-item [67]	
	Depression	Patient Health Questionnaire-2 [68]	
**Veteran** **survey only**			
	Posttraumatic epilepsy screen	US National Institute of Neurological Disorders and Stroke common data element	
	Illness perception	Brief Illness Perception Questionnaire [69]	
**Caregiver** **survey only**			
	Distance from Veteran	NAC Questionnaire [44]	
	Challenging behaviors	Care recipient impairment: problem behaviors subscale [70]	
	Positive aspects of caregiving	Caregiving Uplifts Scale [71]	
	Resilience	Response to Stressful Experiences Scale-4 [72]	
	Caregiver choice	NAC Questionnaire	
	Incident health conditions since starting caregiver	Delgado Survey [73]	
	Payment and financial support	Delgado Survey [73]	
	Loss of self	Loss of Self Scale [74]	
	Caregiver burden	Zarit Burden Interview-12 [50]	

^a^NAC: National Alliance for Caregiving.

#### Ecological Momentary Assessment

EMA data collection will focus on primary outcome variables in this study (eg, health, quality of life), aspects of living with PTE, TBI, and other associated comorbidities, as well as aspects of providing care that are likely to change frequently, such as stress, burden, symptoms, unmet needs for support, and thriving. As most traditional surveys are worded retrospectively, the wording of the questions will be modified to reflect the in-the-moment nature of EMA studies. For example, rather than evaluating all unmet needs, we may ask respondents to report what they currently need. In addition, longer questionnaires lead to a higher perceived burden, lower compliance, and more careless responses [75]; therefore, we will avoid using long questionnaires in EMA. Before beginning the EMA data collection, selected items will be used to pilot the study. We will ask 10 people who are similar to the intended study sample to participate in pilot testing, including a brief interview thereafter. This step will provide reasons for missing or noncompliant reports to adapt reliable and valid measures accordingly. Cronbach α will be used to estimate the internal reliability of the questions and the consistency of respondents’ answers.

Participants will be trained on how to use the software, what questions mean, when is the timing of prompts, what to do if a prompt is missed, and whom to contact for assistance. Previous studies on people with acquired brain injury have demonstrated that EMA is a feasible data collection option in this population. For example, Forster et al [76] reported that among a sample of hospitalized patients with TBI who completed an EMA study of 8 assessments per day for 7 consecutive days, 71.6% of prompts were fully completed. There was no difference in compliance based on age or functional impairment level [76].

#### Qualitative Data

We propose a descriptive design to capture information about the experiences of veterans and caregivers. We will consent ≤40 participants for individual interviews. Semistructured individual telephone interviews will be audio recorded and transcribed. The use of audio recording will facilitate the verbatim transcription of these interviews and, subsequently, content analysis. Each interview will be conducted using an interview guide that will include structured, open-ended questions and probes to stimulate a discussion between the participant and the interviewer. The interviewer will be one of the research team members, and this person will have training and experience in qualitative research. The interviews will be conducted by phone after the interviewer establishes a connection with the participant [77,78]. The first 2 interviews will be used as pilots to assess the content and flow of the conversation. Performing this pilot test will provide the opportunity to modify and add probes based on new information valuable for the aims of this study [79]. Using the secondary data for veterans and a web-based survey, we will incorporate probes and questions of interest associated with the effect of epilepsy, TBI, or other conditions in veterans and caregivers.

Transcripts will be the main source of information during the analysis [78]. Field notes are a secondary data collection method used in qualitative research [80]. After each interview, the interviewer will record observational and methodological field notes. Field notes recording the experience of the interviewer performing the interview are useful to maintain the rigor of the study and when conducting an audit trail of the study activities. Some guiding questions in developing field notes are, “What happened and what was involved?”, “Who (nonidentifiable) was involved?”. “Where did the activities occur?”, and “Why did an incident take place and how did it actually happen?” [81]. Data will be digitally recorded and transcribed by the research assistant for analysis.

### Study Duration

We plan to begin collecting baseline surveys and EMA data from January 2022. Baseline data collection will continue for approximately 9 months. EMA data collection will begin shortly after baseline surveys are launched and will continue for 2 years after baseline data collection is complete, or nearly 3 years in total. Depending on recruitment and saturation, qualitative interviews will begin approximately 6 months after EMA data collection begins and will continue for up to 2 years thereafter. The project will be complete by September 30, 2024.

### Study Status

We have drafted the baseline surveys, EMA measures, and interview guides and have begun working to secure human subject approval. We have started recruiting the Service Member, Veteran, and Caregiver Community Stakeholders Group members.

### Ethical Approval

The University of Utah, Salt Lake City VA, and the University of Texas Health Science Center, San Antonio, approved this study. The study is currently being reviewed by the Human Research Protection Office of the United States Army Medical Research and Development Command. All data with protected health information or personal identifiable information will remain on a server that has federally approved encryption at VA Salt Lake City. Analyses of the deidentified data may occur at other sites.

### Data Analysis

#### Overview

We will conduct cross-sectional analyses using baseline survey data to address aim 1, and we will conduct longitudinal analyses incorporating baseline and follow-up EMA data to address aim 2 and aim 3. In all analyses that incorporate both the veteran and caregiver, we will treat the data as correlated. Below, we outline our analytical approaches for each aim.

#### Aim 1

Describe and compare the caregiver and veteran experiences of post-9/11 veterans with PTE to those of other post-9/11 veterans without PTE.

Using the baseline survey, we will calculate and report means and SDs for continuous measures (eg, hours of care and length of care), as well as percentages and 95% CIs for categorical variables (eg, relationship to veteran, choice in caregiving, and veteran comorbidities; Table 3).

When comparing the health and caregiving characteristics of veterans and their caregivers by PTE status, we will use *t* tests for continuous scores and chi-square tests for categorical variables. For all hypotheses, we will compare veterans with PTE and their caregivers to those with: (1) NTE; (2) TBI only; and (3) neither epilepsy nor TBI. We will also make comparisons adjusting for important covariate information using log-binomial regression models to estimate the prevalence ratio [82].

**Table 3:** Summary of statistical tests to be conducted for hypotheses within specific aim 1.

Hypotheses	Outcome measure	Statistical tests	
		Crude	Adjusted covariates
Veterans with PTE^a^ will report poorer health than veterans with NTE^b^, TBI^c^ only, and neither epilepsy nor TBI.	VR-12^d^ Veteran health	2-tailed *t* test: VR-12 score; *χ*^2^ test: VR-12 ≤45; *χ*^2^ test: presence of comorbidity	Veteran age, sex, SES^e^, social support
Caregivers of veterans with PTE will report poorer health than caregivers of veterans with NTE, with TBI only, and neither epilepsy nor TBI.	VR-12 Caregiver health	2-tailed *t* test: VR-12 score; *χ*^2^ test: VR-12 ≤45	CG^f^ age, sex, SES, social support
Caregivers of veterans with PTE will higher burden than caregivers of veterans with NTE, with TBI only, and neither epilepsy nor TBI.	Zarit 12-item caregiver burden	2-tailed *t* test: Zarit score; *χ*^2^ test: Zarit score ≥16	CG age, sex, SES, social support, health, relationship to Veteran
Caregivers of veterans with PTE will report higher caregiving intensity than caregivers of veterans with NTE, with TBI only, and neither epilepsy nor TBI.	NAC^g^ caregiving intensity level	*χ*^2^ test: intensity category; *χ*^2^ test: intensity ≥4	CG age, sex, SES, social support, health, relationship to Veteran
Caregivers of veterans with PTE and associated comorbidities will report poorer health, higher burden, and higher caregiving intensity than caregivers of veterans with PTE without comorbidities and veterans with similar comorbidities (eg, depression and headache) but without epilepsy.	Modified O’Malley caregiver needs measure	2-tailed *t* test: number of unmet needs; *χ*^2^ test: presence of each unmet need	CG social support and SES

^a^PTE: posttraumatic epilepsy.

^b^NTE: nontraumatic epilepsy.

^c^TBI: traumatic brain injury.

^d^VR-12: veterans RAND-12.

^e^SES: socioeconomic status.

^f^CG: caregiver.

^g^NAC: National Alliance for Caregiving.

#### Aim 2

Evaluate the change in available support and unmet needs for care among caregivers of post-9/11 veterans with PTE over a 2-year period.

To evaluate differences in the unmet needs among caregivers, we will use a 2-tailed *t* test to compare the number of unmet needs among caregivers of veterans with and without PTE at baseline. We will also compare the PTE group and each of the other 3 groups. At follow-up, we will calculate the proportion of caregivers who had the same unmet needs at both time points and determine whether specific needs remained constant over time. To account for potential confounding, we will use a generalized estimating equation model to estimate the likelihood of unmet needs being resolved over time, running models separately for all unmet needs being resolved and for each individual need that is evaluated [83]. We will adjust for changes in caregiver and veteran health (trajectories; described the *Aim 3* section), Veteran comorbidity, and the demographic characteristics of both veterans and their caregivers (age, sex, and socioeconomic position).

Qualitative data will contribute to both aim 1 and aim 2. We will use a descriptive phenomenological approach for qualitative data analysis [81,84]. The analysis will include the following steps:

1. Bracketing and phenomenological reduction: In-depth description of the researcher’s experience with the phenomena. A detailed description of the interviews will be provided. The stories or narrative will exclude any information that may link the information with the identity of the participant (eg, names, specific units, and platoon) [85,86].

2. Development of units of meaning: The list of units of relevant meaning extracted from each interview will be carefully scrutinized and clearly redundant units will be eliminated [81].

3. Clustering of units of meaning to form themes: With the list of nonredundant units of meaning in hand, we will again bracket our presuppositions to remain true to the phenomenon. We will seek to elicit the essence of *meaning units* within a holistic context. Clusters of themes are typically formed by grouping units of meaning together [78,81]. The data will be examined to identify common themes, and extract significant statements to compile a set of themes based on the research question, leading to the creation of *meaning units* and textual descriptions. To achieve this, the transcripts will be read several times, and significant statements will be extracted for closer evaluation. Statements that are similar in meaning will be grouped together, forming *meaning units*.

4. Summarize each interview, validate and modify: A summary that incorporates all the themes elicited from the data provides a holistic context. Reflecting on the transcripts, we will provide a textural description, which is a description of what the participant’s experience is with the phenomenon. The description will include verbatim examples or quotes. In addition, we will use triangulation, which is a method that compares the information captured from interviews and paired with other sources of information [78]. Triangulation will incorporate information from interviews and surveys to identify commonalities and themes.

#### Aim 3

Identify veteran and caregiver characteristics associated with the 2-year health trajectories of caregivers and veterans with PTE compared with veterans without PTE.

To identify health trajectories among both veterans and caregivers, we will use group-based trajectory modeling, a finite mixture modeling approach that estimates the mean trajectories for a given number of groups and for each individual provides a probability of membership to each latent class (group) [87,88]. We will construct a model for veterans and a separate model for caregivers using the traj command in Stata, a user-created plugin available for download, and the associated trajplot command to plot the estimated trajectories. We will estimate these trajectories using only time since the onset of epilepsy as the independent variable and change in health status as the outcome, considering between 1 and 7 different trajectory groups and choosing the best model based on the Bayesian information criterion along with a qualitative assessment of whether each trajectory group identified by the model provides unique information [87]. Individuals will be assigned to the trajectory group for which they have the highest probability of membership (posterior probability). We will characterize trajectory groups based on their shape (eg, increasing, decreasing, and stable health). We will use the recommended approaches to test the assumptions [89] of our model and evaluate the goodness of fit [87].

We will calculate and compare the percentage of veterans in each health trajectory group based on their PTE and TBI status, and repeat this for caregivers, using chi-square tests for comparisons. As in aim 1 and as the sample size allows, we will compare veterans or caregivers of veterans with PTE to those with: (1) NTE and (2) TBI only, and no epilepsy and no TBI.

We will evaluate how demographic characteristics, veteran symptoms, comorbidity, and caregiving characteristics (eg, burden and intensity) vary across groups by calculating the proportion of individuals assigned to each group with a given characteristic. In addition, we will use a generalized estimating equation model to estimate the likelihood of veteran trajectory membership based on caregiver burden and caregiver health trajectory. We will also run more fully adjusted versions of this model to account for veteran and caregiver age, sex, relationship, and socioeconomic status.

**Figure 3:**
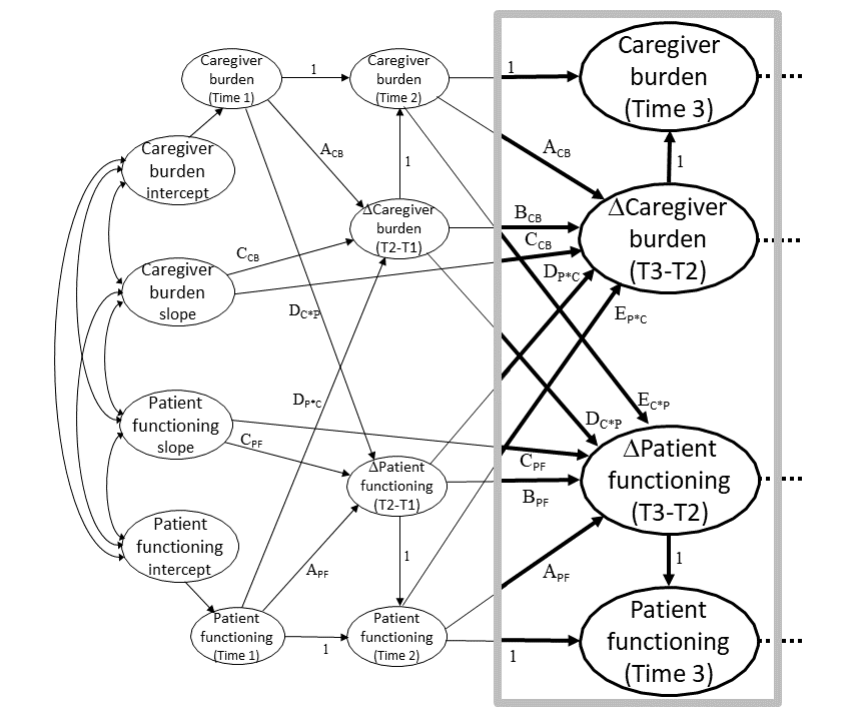
Simplified example of changes-on-changes extension of the bivariate dual latent change model. C: caregiver; CB: caregiver burden; P: patient; PF: patient functioning; T: time.

To examine the degree to which changes in caregiver burden and caregiver intensity are dynamically related to changes in the health trajectory of the care recipient over time, we will use the changes-on-changes extension of the bivariate dual latent change score model [90], a simplified version of which is presented in Figure 3. This specialized structural equation model is particularly useful in examining so-called *chicken and egg* questions about causality, as it allows the changes over time in 2-coupled variables to be modeled as a dynamic system in which each outcome in the model can simultaneously predict and be predicted by the other variable in the system. With this model, changes between assessments for each construct are explicitly modeled (labeled ΔCaregiver Burden and ΔPatient Functioning) at each time point, and changes in each of the 2 outcomes in the model can be predicted as a function of up to 5 predictors, 3 of which emanate *within-construct* and 2 of which emanate *across-constructs* [90]. The highlighted box in the figure represents the within-construct predictors of change and includes the following: (1) a constant change component (arrows designated as *C*), (2) the focal variables prior levels (*A*), and (3) the focal variables prior changes (*B*) [90]. The across-construct predictors, which are typically of most interest, allow a focal variable to be predicted by the following: (1) prior levels of a coupled variable (*D*), and (2) prior changes in a coupled variable (*E*) [90]. To the degree to which prior changes in one variable predict later changes in another, it is said to be a leading indicator of that variable, and it is possible that both variables in a dynamic system are reciprocally related such both are causes and effects of one another. This model can be used to predict complex web-based trajectories owing to the variety of combinations of possible predictors.

#### Missing Data

We will examine several pairs of theoretically related constructs using this approach for the entire sample. Models will be estimated using full information maximum likelihood estimation to account for missing data that may exist. Models will be evaluated using common indexes for structural equation models (eg, comparative fit index and root mean square error of approximation). After establishing the best-fitting dynamic model for a particular pair of grouped variables, we will examine multiple-group models to compare the model fit for PTE dyads versus non-PTE dyads. In addition to the specific full information maximum likelihood estimation strategy for the bivariate dual latent change score model, we will conduct both complete-case analyses, in which only respondents or dyads with complete information on all covariates assessed are included, and we will create imputed data sets using multiple imputation using chained equations [91,92] to retain all participants. We will descriptively compare the characteristics of people with no missing data and those with imputed data to evaluate whether the groups differ systematically or if data appear to be missing at random. We will report the results from both sets of analyses in manuscripts and presentations.

#### Sensitivity Analyses

Across aims, we will conduct sensitivity analyses in which we repeat our analyses using (1) only the primary caregiver (as identified by the veteran); (2) only definite or probable cases of PTE, and combine suspected cases with participants without PTE; and (3) excluding people with epilepsy before combat exposure or TBI.

## Results

The University of Utah approved this study on December 14, 2020. Recruitment will begin once it is approved by the Human Research Protection Office of the United States Army Medical Research and Development Command.

## Discussion

### Principal Findings

The Institute of Medicine identified the evaluation of quality of life in epilepsy as a priority. Concern about the profound impact of epilepsy led to the Institute of Medicine (now the National Academy of Medicine) conducting an in-depth study of the public health dimensions of epilepsy. The study concluded that “comprehensive, timely, and accurate epilepsy surveillance data are needed to provide a better understanding of the burden of epilepsy, its risk factors and *outcomes*, and *health service needs*” [93]. This study will address this priority and the existing gap in the literature related to the needs and health impacts of caregiving for veterans with epilepsy, including both PTE and NTE.

Our long-term goal is to implement the findings of the proposed study in practice to improve the health and well-being of veterans with PTE and epilepsy and their caregivers. We follow the RAND Corporation’s blueprint for MVC research [94] as we develop our strategy. Specifically, of the 10 research objectives identified by their stakeholder panels, we will directly address the following 5 objectives: (1) describe caregivers (and unique characteristics of caregivers of PTE or epilepsy); (2) assess how the needs of care recipients change over time; (3) document the effects of caregiving on care recipient outcomes; (4) document the effects of caregiving on caregiver outcomes; and (5) examine factors associated with caregiver and care recipient harm.

**Figure 4:**
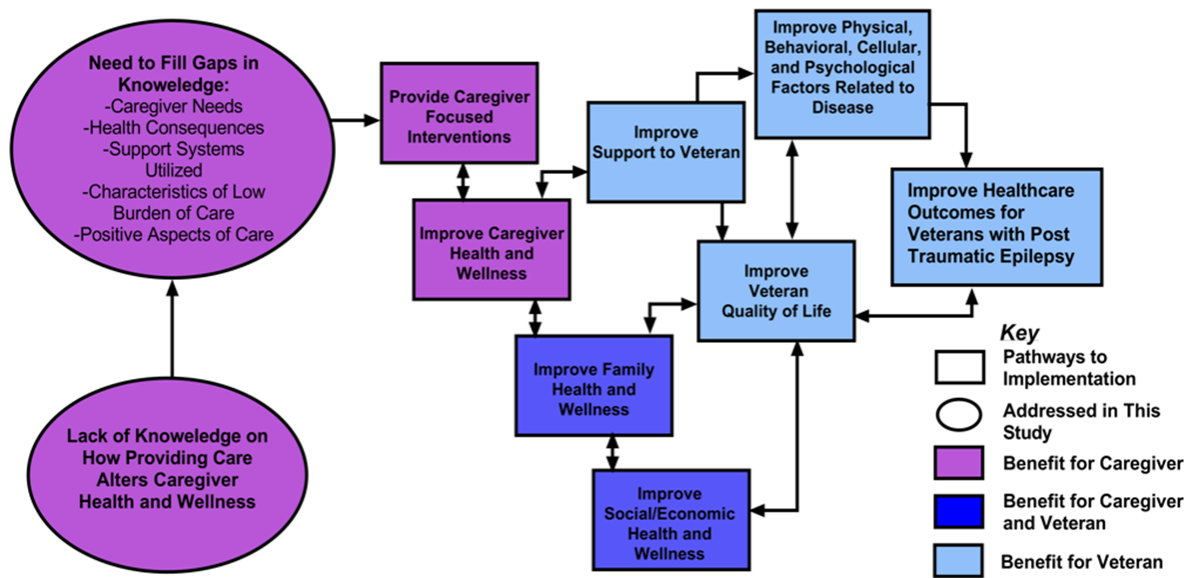
Pathway for implementation.

We also believe that it is critical to plan for implementation as we conduct the research. To this end, we have developed a pathway for clinical implementation and improved health outcomes to guide the work, which considers benefits to both veterans and caregivers. Figure 4 illustrates the process by which we plan to implement the findings of the proposed study into practice to improve the health and well-being of veterans with PTE or epilepsy and their caregivers.

### Strengths

Our baseline survey will include validated measures that have been used in many previous studies and therefore will enable comparisons to other veteran and caregiver populations. In addition, EMA data will enhance our ability to use real time experiences of veterans and their caregivers to identify health outcomes, quality of life, unmet needs, and supports that may inform programs and interventions. By collecting numerous random assessments over a period of time, the EMA provides a representation of how individuals’ experiences and behaviors vary over time and across situations. For this study, the method focuses on symptoms, experiences with caregiving and adaptive behaviors, and aims to map the fluctuations of daily function within both caregivers and care recipients. This method is considered suitable for understanding daily changes in mood, stress, and medical symptomology [38,45,95]. EMA is a useful tool in research for collecting data which are often unobtainable by other methods and providing ecologic validity based on a reduction of recall bias and therefore assessment error. It has also been shown to provide clarity on individual pattern assessments that can otherwise be misunderstood. EMA will allow unprecedented information on unmet needs among MVCs and veterans with PTE.

To our knowledge, our analyses will be novel, as no one has examined longitudinal dyadic data using the changes in the extension of the bivariate dual latent change score model. The partitioning of prior levels and prior changes as distinct predictors of later changes is invaluable in helping us understand the underlying mechanisms that can be targeted for intervention. In addition, the study includes a broad range of measures that represent physical, mental, and emotional health of dyads. These data will enable us to not only evaluate individual-level impact on key outcomes but also to better understand the bidirectional health-related influences between veterans and caregivers. We expect that interventions designed to leverage relational strengths or address interpersonal barriers will ultimately result in better outcomes for both care recipients and caregivers [96-98].

### Limitations

Study limitations include the fact that the sample may not represent all geographic regions of the United States or all subgroups of veterans. In addition, whereas the study is longitudinal, follow-up will occur over a 2-year period, and therefore, we will not be able to assess the veterans’ and caregivers’ experiences over a longer period of time. As noted in the methods, our sample size may be too small to compare all 4 study groups on some measures, including health trajectory groups, depending on the distribution of responses and experiences. To keep the survey length manageable, we used screening versions of validated questionnaires for anxiety and depressive symptoms and therefore do not have more detailed measures of these experiences.

### Conclusions

This study will fill a critical gap in the literature related to the needs, activities, and positive aspects of veterans with PTE and their caregivers. It will also strengthen our understanding of the relationship and positive aspects of caregiving for veterans with TBI and other injuries. We have designed the study using integrative frameworks and are leveraging a multidisciplinary team to use this work as the foundation for future interventions that will strengthen caregiver resilience and improve the health and quality of life of post-9/11 veterans and their family members who support them.

## Abbreviations

BRFSS: Behavioral Risk Factor Surveillance System
EMA: ecological momentary assessment
mTBI: mild traumatic brain injury
MVC: military and veteran caregiver
MVCHW: Military and Veteran Caregiver Health and Well-being
NTE: nontraumatic epilepsy
PTE: posttraumatic epilepsy
TBI: traumatic brain injury
VA: Department of Veterans Affairs
VPES: Veterans Posttraumatic Epilepsy Study
